# Bullying and Cyberbullying Are Associated with Inappropriate Use of the Internet, Cell Phones, and Video Games in Children and Adolescents

**DOI:** 10.3390/ejihpe15050082

**Published:** 2025-05-15

**Authors:** José Enrique Moral-García, Alba Rusillo-Magdaleno, Fredy Alonso Patiño-Villada, Emilio J. Martínez-López

**Affiliations:** 1 Department of Didactics of Musical, Plastic and Corporal Expression, Faculty of Humanities and Education Science, University of Jaén, 23071 Jaén, Spain; jemoral@ujaen.es (J.E.M.-G.); emilioml@ujaen.es (E.J.M.-L.); 2 University Institute of Physical Education and Sports, University of Antioquia, Medellín 050010, Colombia; fredy.patino@udea.edu.co

**Keywords:** school bullying, adolescents, perpetration, cyberbullying, internet, cell phone, video games, social networks, victimization

## Abstract

The aim of the present study was to analyze the association of bullying and cyberbullying with the level of Internet, cell phone, and video game use in children and adolescents. In total, 677 Spanish students (53.03% girls) aged 10 to 16 years (13.81 ± 1.56) participated. The association between variables and risk of exposure was carried out by analysis of covariance (ANCOVA) and binary logistic regression (odds ratio = OR), respectively. The effects of both victimization and perpetration in bullying and cyberbullying were analyzed separately to identify differences by role. All analyses were performed separately for boys and girls and adjusted for age, body mass index, mother’s education, and average weekly physical activity. The results showed that both victims and perpetrators of bullying and cyberbullying present a significant increase in and risk of abusive and inappropriate use of the Internet, cell phones, and video games. Girls involved in bullying/cyberbullying behaviors reached the highest levels of inappropriate use of the Internet, cell phones, and video games with respect to peers not affected by bullying behaviors. In all cases, girls, both victims and perpetrators of bullying and cyberbullying, multiplied the risk of harmful use of these devices by at least 3 times. It is suggested to implement educational policies to prevent situations, especially cyberbullying, in both victims and perpetrators, prioritizing student safety.

## 1. Introduction

In today’s society, Internet use among children and adolescents has increased considerably in recent years due to the easy access and expansion of technology (Barbieri et al., 2024; Ciacchini et al., 2023). Several studies have concluded that between 65% and 78% of children and young people, respectively, consume the Internet excessively on a daily basis (Do et al., 2020; Pons-Salvador et al., 2022), and addiction to cell phones now affects all social strata, with 15% of young people spending more than four hours a day online (Radesky et al., 2020). This abuse in the use of mobile devices has aroused scientific interest due to the negative impact it has on physical, emotional, and social health (Arul-Prasath et al., 2022). At the physiological level, it has been shown that the overuse of electronic devices leads to structural changes in the brains of young people (Wacks & Weinstein, 2021; Hou et al., 2022) as impairment in cognitive control during emotional processing (Fabio et al., 2022; Hadlington, 2015) and reduction in functional connectivity in brain regions related to cognitive control of emotional stimuli (Anbumalar & Binu Sahayam, 2024; Chun et al., 2018). On the other hand, the excessive consumption of video games in young people is associated with significant risks of low psychological well-being (Feng, 2022), poor nutrition (Puolitaival et al., 2020), anxiety (Hou et al., 2022), social isolation, and depression (F. Craig et al., 2021). Despite the above, some video games, especially those with an interactive design, can promote physical activity and the development of social and cognitive skills (Moller et al., 2023). In addition, factors such as the intrinsic and extrinsic motivation of young people can mediate the frequency of video game use due to the usefulness of these technologies and their integration in learning activities (Camilleri & Camilleri, 2020).

According to recent studies, the excessive use of the Internet, mobile devices, and video games also impairs the cognitive control of young people and reduces their ability to manage emotions, considered key factors for maintaining healthy social relationships (Lesinskienė et al., 2024). Another common aspect of the overuse of these technological applications is their relationship with the appearance of bullying behaviors or attitudes in children and adolescents, such as bullying and cyberbullying (Ak et al., 2022; Cagirkan & Bilek, 2021). It seems that young people take refuge in new technologies to avoid situations of harassment or bullying because the virtual environment provides a temporary escape from reality (Feijóo et al., 2021; Nickerson, 2019).

Although many studies consider that the abuse of technology can contribute to the development of bullying behavior, it is essential to consider the opposite effect as well. The consequences of bullying, considered as a manifestation of mistreatment between students, characterized by acts of physical or mental violence and maintained over time (Nain et al., 2023), could generate a rebound effect by further increasing the time spent on the Internet, using cell phones, and playing video games (Serrano et al., 2023). Moreover, the repercussions of these bullying behaviors affect victims, as well as perpetrators and observers, manifesting themselves in behaviors of impulsivity, anger, and school violence (Bourou & Papageorgiou, 2023; Simion, 2023) that have a very negative influence on the educational environment of adolescents (Al-Turif & Al-Sanad, 2023; Iñiguez-Berrozpe et al., 2021; Soto-García et al., 2024). Cyberbullying, on the other hand, is characterized by its persistent nature and the ease with which perpetrators can hide their identity (Al-Turif & Al-Sanad, 2023) and has more long-term implications for the students involved, such as low self-esteem (Tsaousis, 2016), mental health problems (Kirkbride et al., 2024), low social development (Nasti et al., 2023), and poor academic performance (L. Li et al., 2020; Riffle et al., 2021).

Recent studies have also revealed that young people may respond differently to the above stimuli depending on their age (Kim et al., 2021), body mass index [BMI] (Xu et al., 2020), weekly physical activity (Gmmash et al., 2023), and their parents’ level of studies (Jensen et al., 2023). A high BMI has been linked to a higher risk of victimization and lower self-esteem, which could favor greater dependence on online activities (Kim et al., 2021). Likewise, regular physical activity is related to greater psychological well-being and may act as a protective factor against the problematic use of technologies and involvement in bullying situations (Gmmash et al., 2023). Finally, maternal educational level is considered a predictor of cognitive development and self-regulation in adolescence, variables that influence both involvement in bullying behaviors and the responsible use of digital devices (Jensen et al., 2023; Choudhary et al., 2024). In addition, it appears that adolescents of different sexes tend to experience and engage in bullying unequally (Smith & Berkkun, 2017). While boys are more likely to engage in direct and physical forms of bullying, girls are more likely to engage in relational bullying such as social exclusion and rumor spreading (Slonje et al., 2013). Girls may also be more vulnerable to cyberbullying due to their greater concern for appearance and social acceptance, which makes them more susceptible to virtual psychological attacks (Frisén & Berne, 2020). On the other hand, boys may pressure their peers to demonstrate their dominance and power through aggressive online behavior (Zsila et al., 2019). These dynamics, together with differential access to and use of technologies, contribute to the fact that the experiences and consequences of bullying and cyberbullying vary significantly between sexes.

Numerous studies have addressed the association of school bullying and cyberbullying with the abuse of Internet and cell phone use (Camilleri & Camilleri, 2020; Feng, 2022; Foerster et al., 2019; Unni & Prasanna Kumar, 2023) and video games (F. Craig et al., 2021; Hou et al., 2022; Puolitaival et al., 2020). However, the explicit association of victimization and perpetration with the excessive use of these technologies has been little explored (Mumford et al., 2023). The present research provides a pioneering approach in differentiating the roles of bullying, which may offer new insights into how both victims and perpetrators of bullying may overuse and addictively use new technologies (Lozano-Blasco et al., 2022). Based on the above, the aim of the present study was to analyze the possible association of bullying and cyberbullying with the use of the Internet, cell phones, and video games in the Spanish school and adolescent population of both sexes, after adjusting for age, BMI, mother’s level of education, and average physical activity. We also sought to determine the level of risk involved in bullying victimization/perpetration and cyberbullying in relation to abusive use of the Internet, cell phones, and video games. We hypothesized that those young people with a higher level of participation in bullying and/or cyberbullying, regardless of their role, would in turn have higher levels of inappropriate Internet, cell phone, and video game use.

## 2. Materials and Methods

### 2.1. Participants

A total of 677 primary and secondary school students aged 10–16 years (mean age ± standard deviation: 13.81 ± 1.56 years) (53.03% girls and 46.97% boys) participated in the present cross-sectional quantitative study. Data collection took place from February to May 2023. Seven educational centers in various provinces of Andalusia were studied, of which three were publicly owned and four subsidized—three rural and four urban centers of a medium socioeconomic level. The sample was selected by convenience. The anthropometric and sociodemographic characteristics are detailed in Table 1.

### 2.2. Predictor/Independent Variables

Bullying and cyberbullying

The level of bullying was assessed using the “European bullying intervention project questionnaire” instrument, Spanish version, from Ortega-Ruiz et al. (2016), 14 items. To assess cyberbullying, we used the Spanish version of the “European cyberbullying intervention project questionnaire” (ECIPQ; Del Rey et al., 2015) which includes 22 items. Reliability scores were high for both bullying (Crombach’s α victimization = 0.830 and Crombach’s α perpetration = 0.811) and cyberbullying (α cybervictimization = 0.821 and α cyberperpetration = 0.837). Both questionnaires were administered individually and employ a Likert-type scale with a score ranging from 1 = never to 5 = more than once a week. The items explore the frequency with which the described behaviors have occurred during the past two months, and both required approximately 15 min to complete.

### 2.3. Dependent Variables

Internet, cell phone, and video game use

The “Spanish PIUQ-9” instrument was used to quantify the level of Internet, cell phone, and video game use (Trisano et al., 2022), and it was designed to assess the psychological and behavioral impact of problematic Internet use along three main dimensions (obsession, neglect, and control disorder). “SAS-SV” (Kwon et al., 2013) was used to measure the degree of dependence on cell phones and assess how it affects students’ emotional attachment, compulsion, withdrawal symptoms, and difficulty in reducing bullying, and, to assess the degree of dependence on cell phones, “Internet Gaming Disorder Scale–Short–Form (IGDS9–SF)” was used (Pontes & Griffiths, 2015). The latter measures dependence symptoms in online video game players by assessing problematic behaviors and experiences related to Internet gaming. Each instrument included 9 items and used a Likert-type scale, with scores ranging from 1 = never/totally disagree to 5 = almost always/always/totally agree, with the highest score being the maximum level of Internet, smartphone, and video game use addiction in the past year. All questionnaires were administered individually and presented high levels of reliability—Internet use (Crombach’s α = 0.831), cell phone use (Crombach’s α = 0.849), and video game use (Crombach’s α = 0.813).

### 2.4. Confounding Variables

Age, body mass index, mother’s education, and average weekly physical activity

The age and educational level of the mother of each participant were recorded by means of a sociodemographic data questionnaire. Age was considered a confounding variable given its relevance in previous studies, where it has been shown that maturational and emotional development, together with psychological factors, significantly influence the management of stress associated with bullying. Similarly, the maternal educational level has been related to cognitive development and self-regulation in adolescents, which may affect both their involvement in bullying behaviors and their use of digital technologies (Barlett et al., 2021; Geng et al., 2022; Choudhary et al., 2024; Yang et al., 2023). BMI was calculated using the Quetelet formula: weight (kg)/height^2^ (m). To obtain weight and height measurements, an ASIMED^®^ type B, class III digital scale and a SECA^®^ 214 portable measuring rod (SECA Ltd., Hamburg, Germany) were used. Both measurements were taken in light clothing and without footwear. The level of weekly physical activity was assessed using the “PACE+ Adolescent physical activity measure physical” questionnaire (Prochaska et al., 2001). This consists of two items asking the number of days in which the participants have performed at least 60 min of physical activity at moderate or vigorous intensity during the last 7 days and during a typical week. The final score was obtained by averaging both responses (P1 + P2)/2). Its reliability index was α = 0.744. Both BMI and weekly physical activity were considered since they are related to physical and mental well-being, as well as to students’ learning and self-esteem (Jaiswal et al., 2020; Kim et al., 2021).

### 2.5. Procedure

Data collection was conducted during the 2022/2023 academic year. The purpose and nature of the study were communicated verbally and in writing to students, parents, and legal guardians. Authorization was obtained from the school administration and physical education teachers. All the students involved were informed, prior to filling out the questionnaires, about the concepts of bullying and cyberbullying, including examples of behaviors that can be identified with these forms of harassment. In order to preserve confidentiality and anonymity, the names of the participants were coded. During the completion of the questionnaires and weight and height measurements, a specialized researcher gave the instructions and controlled the time, while two research assistants observed possible doubts and any possible disturbances (e.g., separation space to guarantee the confidentiality of the answers, prevent noise outside the classroom, avoid confusing students, operate electronic tools, and provide an Internet connection). The estimated time to complete all questionnaires, relative to the dependent and independent variables, was approximately 15 min. This study was approved by the Bioethics Commission of the University of Jaén (Spain), reference NOV.22/2.PRY. The design took into account the current Spanish legal regulations governing clinical research in humans (Royal Decree 561/1993 on clinical trials), as well as the fundamental principles established in the Declaration of Helsinki (2013, Brazil).

### 2.6. Statistical Analysis

Comparison of the continuous and categorical variables between boys and girls was carried out using Student’s *t* test and χ^2^ tests, respectively. The normality and homoscedasticity of the data were verified using the Kolmogorov–Smirnov and Levene tests, respectively. To study whether adolescents who had never experienced bullying and cyberbullying victimization/perpetration were more likely to abuse the Internet, cell phones, and video games than those who had been victims/perpetrators, an analysis of covariance (ANCOVA) was performed. Measures of Internet, cell phone, and video game use were used as dependent variables, and bullying victimization, bullying perpetration, cyberbullying victimization, and cyberbullying perpetration were introduced as fixed factors. The bullying and cyberbullying values were dichotomized such that participants who stated that they had never been a victim/offender of bullying and/or cyberbullying (questionnaire score = 1) were labeled as “Never”, and those who had ever been a victim/offender (questionnaire score = 2–5) were labeled as “Sometimes”. For the above categorization, the specific nature of the phenomenon of bullying has been taken into account, where it cannot be assumed that an occasional score (e.g., “sometime”) indicates the absence of victimization or aggression. It has been considered relevant to highlight any involvement, even if occasional, as even isolated events can have a significant impact on the psychological and social well-being of minors (Nickerson, 2019).

Because many comparison groups had different sample sizes, effect sizes were calculated using Hedges’ ğ, where 0.2 = small effect, 0.5 = medium effect, and 0.8 = large effect (Martínez-López et al., 2017). The percentage of difference between groups was calculated as [(Large-measurement − small-measurement)/small-measurement] × 100. To determine the level of risk of bullying victimization/perpetration and cyberbullying as having lower values in the level of Internet, cell phone and video game use, a binary logistic regression was carried out. For this purpose, the dependent variables were dichotomized, taking the median as a reference (Kobel et al., 2022; Zhong et al., 2023). Each strategy was classified as high ≥ median (reference group) vs. low < median (risk group). In addition to the dichotomous categorization of participants as “Never” vs. “Sometimes” involved in bullying/cyberbullying, an additional classification was introduced to distinguish the frequency of involvement. Following prior prevalence studies in Spanish school populations (Feijóo et al., 2021; W. Craig et al., 2020), participants were classified as frequent if they reported involvement “once a week” or “more than once a week” (Likert score ≥ 4) and as occasional if they reported involvement “occasionally” or “once or twice a month” (Likert score = 2 or 3). This distinction was applied separately for both victimization and perpetration in traditional bullying and cyberbullying.

In all analyses, age, BMI, mother’s educational level, weekly physical activity, and academic performance were used as covariates. Analyses were performed separately for boys and girls. A 95% confidence level (*p* < 0.05) was used for all results. All calculations were performed with the statistical program SPSS, v. 25.0 for WINDOWS (SPSS Inc., Chicago, IL, USA).

## 3. Results

### General Descriptive Analysis

As previously detailed in the Methods section, the participants were further classified as frequent or occasional according to their responses to the questionnaires. Frequent involvement was considered to be that for whom frequency was once a week or more (Likert score ≥ 4), and occasional involvement was considered to be less than once a week (scores 2–3).

In relation to traditional bullying victimization, 82.3% of the student body indicated that they had been victims at some time. Of these, 7.7% were classified as frequent victims, while 74.6% were occasional victims. Only 17.7% stated that they had never been assaulted. Regarding perpetration, 4.3% were frequent offenders and 65.9% were occasional offenders, compared to 29.8%, who reported having never been bullied. In terms of sexes, boys showed slightly higher percentages of frequent aggressive behaviors (3.2% once a week vs. 1.9% of girls).

On the other hand, in the case of cyberbullying, 2.6% of the student body were frequent victims, compared to 52.9% of occasional victims, while 44.5% reported never having suffered it. Cyberbullying perpetration was referred to by 3.4%, who were frequent offenders, and 43.1%, who were occasional offenders. Girls showed a higher prevalence in occasional cyberbullying victimization (51.7%) compared to boys (45.3%).

**Covariance analysis of victimization and perpetration in bullying and cyberbullying with respect to internet use.**

**Figure 1:**
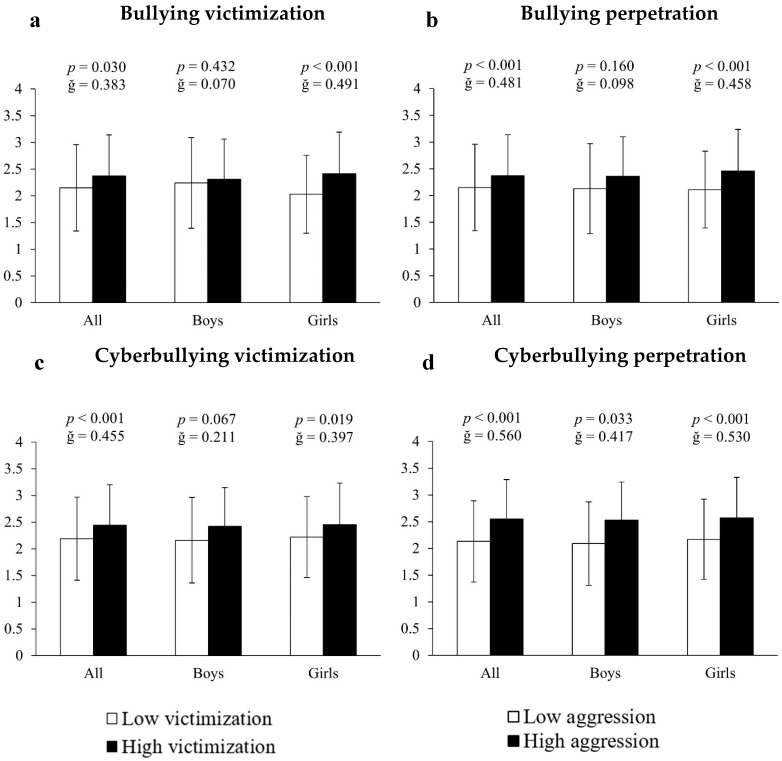
Association of victimization and perpetration in bullying and cyberbullying with respect to internet use.

Analysis of covariance employing inappropriate internet use as the dependent variable and bullying measures as the fixed factor showed that, in all cases, victims and perpetrators of bullying, as well as victims and perpetrators of cyberbullying, presented significantly higher scores (10.23%, 13.68%, 11.42% and 19.72%, respectively) of internet abuse compared to all other participants (all F[1,661] > 11.213, *p* < 0.030, ğ > 0.383, Figure 1a–d). When the results were differentiated by sex it was found that, in all cases, girls immersed in bullying situations had significantly higher scores of inappropriate internet use: (A) for bullying victims = 18.72% (2.41 ± 0.78 vs. 2.03 ± 0.73 a.u., F[1,351] = 13.351, *p* < 0.001, ğ = 0.491, 1 − β = 0.954, Figure 1a); (B) for bullying perpetrators = 16.59% (2.46 ± 0.78 vs. 2.11 ± 0.72 a.u., F[1,351] =16.242, *p* < 0.001, ğ = 0.458, 1 − β = 0.980, Figure 1b); (C) for cyberbullying victims = 10.36% (2.45 ± 0.78 vs. 2.22 ± 0.76 a.u., F[1,351] = 14.609, *p* < 0.019, ğ = 0.397, 1 − β = 0.927, Figure 1c) and (D) for cyberbullying perpetrators = 18.43% (2.57 ± 0.76 vs. 2.17 ± 0.75 a.u., F[1,351] = 27.807, *p* < 0.001, ğ = 0.530, 1 − β = 0.999, Figure 1d). For boys, the results showed 21.05% more inappropriate internet use in cyberbullying perpetrators (2.53 ± 0.71 vs. 2.09 ± 0.78 a.u., F[1,304] = 21.894, *p* = 0.033, ğ = 0.5876, 1 − β = 0.967). No significant differences were found in either victims or victims/offenders of bullying (all *p* > 0.05).

**Covariance analysis of victimization and perpetration in bullying and cyberbullying with respect to cell phone use.**

**Figure 2:**
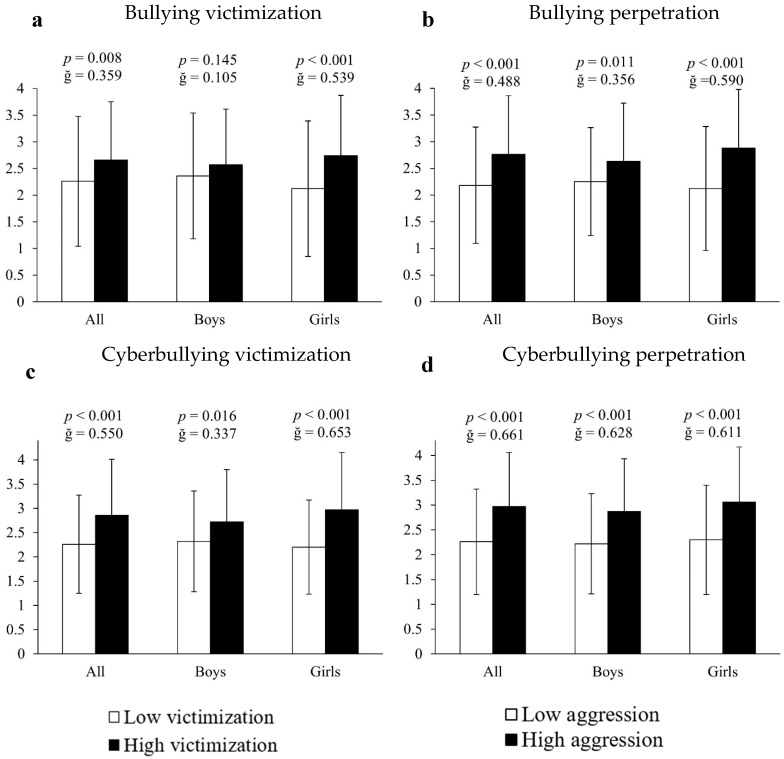
Association of victimization and perpetration in bullying and cyberbullying with respect to cell phone use.

The analysis of covariance employing inappropriate or unhealthy cell phone use as the dependent variable and bullying measures as the fixed factor showed that, in all cases, victims and perpetrators of bullying, as well as victims and perpetrators of cyberbullying, presented higher indicators of cell phone abuse (26.55%, 26.61%, 26.55% and 31.42%, respectively) compared to all other participants (all F[1,661] > 14.363, *p* < 0.009, ğ > 0.358; Figure 2a–d). Results segmented by sex showed that, in all cases, girls involved in bullying contexts had significantly higher scores of inappropriate cell phone use: (A) bullying victims = 29.25% (2.74 ± 1.13 vs. 2.12 ± 1.17 a.u., F[1,351] = 14.171, *p* < 0.001, ğ = 0.539, 1 − β = 956, Figure 2a); (B) bullying perpetrators = 35.85% (2.88 ± 1.11 vs. 2.12 ± 1.16 a.u., F[1,351] = 35.474, *p* < 0.001, ğ = 0.590, 1 − β = 0.999, Figure 2b); (C) cyberbullying victims = 35% (2.97 ± 1.15 vs. 2.2 ± 1.1 a.u., F[1,351] = 56.333, *p* < 0.001, ğ = 0.653, 1 − β = 0.999, Figure 2c) and (D) cyberbullying perpetrators = 33.04% (3.06 ± 1.11 vs. 2.3 ± 1.1 a.u., F[1,351] = 46.874, *p* < 0.001, ğ = 0.611, 1 − β = 0.991, Figure 2d). For their part, boys immersed in bullying situations manifested significantly more inappropriate cell phone use: (A) bullying perpetrators = 16.89% (2.63 ± 1.09 vs. 2.25 ± 1.01 a.u., F[1,304] = 9.497, *p* = 0.011, ğ = 0.357, 1 − β = 0.719, Figure 2b); (B) cyberbullying victims = 14.01% (2.64 ± 1.04 vs. 2.27 ± 1.08 a.u., F[1,304] = 9.753, *p* = 0.016, ğ = 0.337, 1 − β = 0.845, Figure 2c) and (C) cyberbullying perpetrators = 29.28% (2.87 ± 1.09 vs. 2.22 ± 1.01 a.u., F[1,304] = 28.272, *p* < 0.001, ğ = 0.628, 1 − β = 0.979, Figure 2d). However, no significant differences were found in boys who were victims of bullying (*p* > 0.05).

**Covariance analysis of bullying and cyberbullying victimization with respect to video game use.**

**Figure 3:**
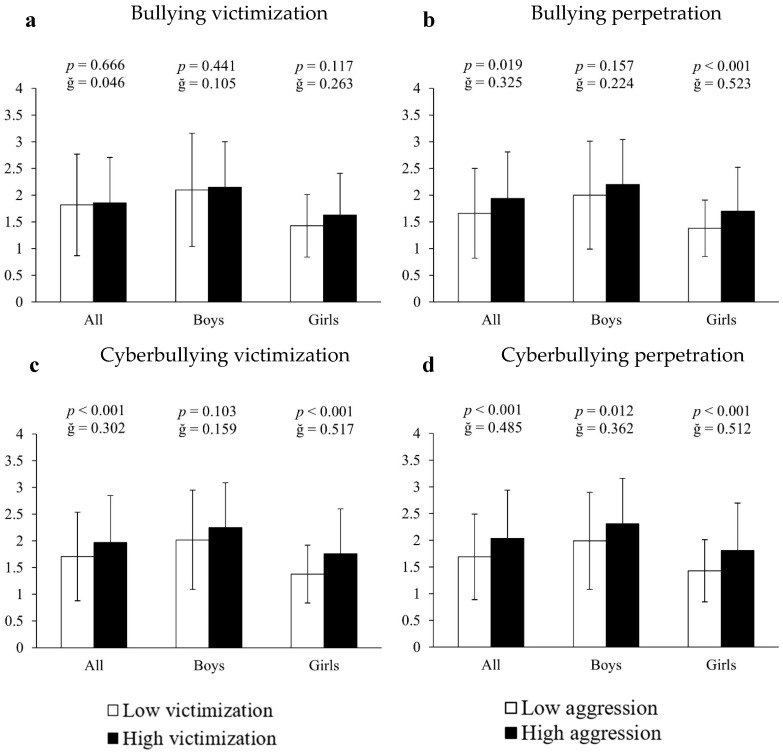
Association of victimization and perpetration in bullying and cyberbullying with respect to the use of video games.

Analysis of covariance using inappropriate or unhealthy video game use as the dependent variable and bullying measures as the fixed factor showed that both bullying perpetrators and cyberbullying victims and offenders had higher values of video game abuse (16.87%, 15.20% and 20.71%, respectively) compared to the rest of the participants (all significant: F[1,661] > 13.158, *p* < 0.020, ğ > 0.301; Figure 2b–d). Sex-segmented analysis revealed inappropriate video game use in: (A) bullying perpetrators = 23.19% (1.7 ± 0.82 vs. 1.38 ± 0.53 a.u., F[1,351] = 24.315, *p* < 0.001, ğ = 0.523, 1 − β = 0.945, Figure 3b); (B) cyberbullying victims = 27.54% (1.7 ± 0.82 vs. 1.38 ± 0.53 a.u., F [1,351] = 26.416, *p* < 0.001, ğ = 0.517, 1 − β = 0.979, Figure 3c) and (C) cyberbullying perpetrators = 26.57% (1.81 ± 0.89 vs. 1.43 ± 0.58 a.u., F[1,351] = 27.916, *p* < 0.001, ğ = 0.512, 1 − β = 0.966, Figure 3d). In boys, inappropriate video game use was observed in cyberbullying perpetrators = 16.08% (2.31 ± 0.85 vs. 1.99 ± 0.91 a.u., F[1,304] = 6.424, *p* = 0.012, ğ = 0.362, 1 − β = 0.715, Figure 3d), but not between victims and perpetrators of bullying and victims of cyberbullying (all *p* > 0.05, Figure 3a–c).

**Binary logistic regression on bullying and cyberbullying victimization and perpetration with respect to internet, cell phone and video game use.**

The data showing the risk of exposure to bullying and cyberbullying (victimization and perpetration) with respect to internet, cell phone and video game abuse are presented in Table 2. Overall, bullied schoolchildren were shown to have 1.60 and 2.07 times the risk of inappropriate use of the internet (OR = 1.606; *p* < 0.001) and cell phone (OR = 2.017; *p* < 0.001) than those who were not bullied, respectively. Bullied girls had a higher risk of abusing the internet (OR = 2.080; *p* < 0.001), cell phone (OR = 2.898; *p* < 0.001) and video games (OR = 1.767; *p* < 0.001). On the other hand, bullying perpetrators expressed 2.11, 2.52 and 3 times more risk of inappropriate use of internet, cell phone and video games, respectively (all *p* < 0.001). Both bullying boys and bullying girls were more likely to have unhealthy internet (OR = 1.503; *p* = 0.027 and OR = 3.826; *p* < 0.001, respectively) and cell phone use (OR = 1.659; *p* = 0.006 and OR = 8.068; *p* < 0.001, respectively). On the other hand, the risk of inappropriate use of video games was increased 3.40 times in bullying girls (*p* < 0.001) but not in boys (*p* > 0.05).

The cyberbullying data indicated that cyberbullying victims had a 4.53, 7.98 and 4.61 times higher risk of misusing the internet compared to those who did not suffer cyberbullying. According to gender, in boy and girl victims of cyberbullying, there was a higher probability of abusive use of the internet (OR = 3.279; *p* = 0.006 and OR = 5.998; *p* < 0. 001, respectively), cell phone (OR = 4.585; *p* = < 0.001 and OR = 16.473; *p* < 0.001, respectively) and video games (OR = 3.999; *p* = 0.049 and OR = 5.484; *p* < 0.001, respectively) compared to those who were not bullied. Furthermore, it was detected that schoolchildren perpetrators of cyberbullying had a significant risk of making unhealthy use of internet (OR = 5.782; *p* < 0.001), cell phone (OR = 14.367; *p* < 0.001) and video games (OR = 3.839; *p* < 0.001). This high probability of risk was confirmed in both boy and girl perpetrators with respect to non-perpetrators (all *p* < 0.001).

## 4. Discussion

The aim of the present study was to analyze the association of bullying and cyberbullying with the use of the Internet, cell phones, and video games in children and adolescents between 10 and 16 years of age. In general, the results showed that both victims and perpetrators of bullying and cyberbullying have higher percentages and a higher risk of abusive use of the Internet, cell phones, and video games than their unaffected peers. In all cases, girls, both victims and perpetrators of bullying and cyberbullying, multiply the risk of harmful use of the Internet, cell phones, and/or video games at least ×3. In both sexes, cyberbullying has a greater negative impact on the abusive use of the Internet, cell phones, and/or video games than traditional bullying. More specifically, the highest risk values are observed in perpetrator boys, who multiply the risk by up to 8.4, and victim girls, who multiply the risk by 16.5 times.

According to the results of this study, young people affected by bullying, both victims and perpetrators, abuse the Internet more (10.2% in both cases) than those who are not affected by bullying. In addition, inappropriate use of the Internet is more prevalent in girls who are victims and perpetrators of bullying (18.7% and 16.6%, respectively) than in boys. Similar results were found by Feijóo et al. (2021), who analyzed the relationship between bullying and cyberbullying and Internet and cell phone use in a sample of 3188 young people aged 12–17 years, as well as those found by Sittichai and Smith (2020), who studied the effect of high levels of Internet use on mental well-being in 1140 students aged 12–18 years. Both studies concluded that both bullying and cyberbullying appear to be associated with abusive and problematic Internet use, as well as directly affecting the mental well-being of adolescents. Regarding gender, some studies have associated bullying and cyberbullying with problematic Internet use more in girls than in boys (Blinka et al., 2023; W. Craig et al., 2020). The present study also found that, although the results are significant in both boys and girls, the risks are higher in girls. For example, in bullying and cyberbullying perpetration, the risks amount to 8.07 and 14.33, respectively.

Furthermore, our results have also revealed that both victimization and perpetration, related to cyberbullying, have a higher negative impact (11.42% and 19.72%, respectively) and twice the risk of abusive Internet use than those affected only by traditional bullying. According to these findings, recent research suggests that cyberbullying has negative associations with respect to Internet use, not as clearly evident in traditional bullying (W. Craig et al., 2020; J. Li et al., 2023). However, some previous research also considers the use of the Internet as a cause of cyberbullying and not as a consequence (Gohal et al., 2023; Mirza, 2020; Uludasdemir & Kucuk, 2019). It appears that cyberbullying, due to its pervasive and anonymous nature, may have a more profound impact than traditional bullying, due to the ease of access to the Internet that technologies provide and the association of Internet use with the search for emotional comfort or support on digital platforms (Huang et al., 2021; J. Li et al., 2023; Richard et al., 2021).

On the other hand, our results reveal that students who are victims of cyberbullying are up to four times more likely to abuse the use of cell phones. Several previous studies have also concluded that students who suffer bullying and cyberbullying have a higher inappropriate use of cell phones than those not involved in bullying situations (Méndez et al., 2020; Shin & Kim, 2023). According to our results, gender emerges as a differentiating factor since female victims have a higher risk (×2.9 for bullying and ×16.5 for cyberbullying) of inappropriate use of cell phones. These findings coincide with those obtained by Feijóo et al. (2021), who found that, in young people aged 12–17 years, girls affected by bullying or cyberbullying abuse cell phones to a greater extent than boys. It seems that girls, both victims and perpetrators, have a greater addiction to social networks than boys and therefore a greater dependence on the use of digital devices such as cell phones (Festl & Quandt, 2016).

Regarding the type of bullying, data have shown that the main associations with inappropriate use of video games are found in victims (15.2%) and perpetrators (20.7%) of cyberbullying. Previous studies have revealed that young people involved in cyberbullying are those who present a greater abuse of video games (Huang et al., 2021) or online games (Richard et al., 2021). In terms of gender, we found a greater addiction to video games among girls who were victims of cyberbullying (27.54%) and perpetrators of bullying (23.19%) and cyberbullying (26.57%). However, other recent research has attributed greater use of video games among boys (Huang et al., 2021; J. Li et al., 2023; Richard et al., 2021) and even observed no differences by sex (Feijóo et al., 2021).

Finally, another aspect that can generate controversy is the differentiating role between victims and perpetrators in the use of video games. In general, there seems to be a consensus that being a victim or perpetrator of bullying or cyberbullying affects screen time (Richard et al., 2021; Shin & Kim, 2023; Sittichai & Smith, 2020). The data presented in this study reveal that the most negative associations of bullying and cyberbullying, towards the abusive use of video games, occur in perpetrators. These findings coincide with those of Teng et al. (2020), whose study, carried out on 3707 adolescents aged 12–19 years, showed that exposure to video games had a significant positive association with perpetration behaviors in bullying and cyberbullying. Therefore, exposure to video games would in turn be associated with a greater likelihood of perpetrating cyberbullying. Although the significant differences between victims and perpetrators are not always evident (Huang et al., 2021; Méndez et al., 2020), cyberbullying perpetrators appear to show the highest association with psychological problems linked to excessive video game use compared to victims (Kotozaki et al., 2023).

### Limitations and Strengths

The study has limitations, such as the impossibility of establishing causality due to its cross-sectional design and the dependence on the sincerity of the participants’ responses, which could have been biased. In addition, convenience sampling limits its representativeness. However, the use of anonymity techniques, reliable and valid instruments, a rigorous collection process, and the inclusion of key covariates (age, BMI, maternal education level, and physical activity) are noteworthy, providing novel results in educational research. Finally, despite the above, and although valid and reliable instruments have been used for this study, the results should be interpreted with caution because the categorization of the participants was based on subjective criteria.

## 5. Conclusions

The present study concludes that, in general, both victims and perpetrators of bullying and cyberbullying show significantly higher values of inappropriate use of the Internet, cell phones, and video games than their classmates not affected by bullying behaviors. Girls involved in bullying/cyberbullying behaviors reach the highest percentages of inappropriate use of the Internet (≥10.36%), cell phones (≥29.25%), and video games (≥23.19%). In all cases, girls, both victims and perpetrators of bullying and cyberbullying, multiply the risk of harmful use of these devices by at least ×3. It was also found that the highest risk values were observed in cyberbullying behaviors, where the perpetrator boys multiply the risk by 8.4 times and victim girls by 16.5 times. Finally, in young people involved in bullying behaviors, excessive and inappropriate use of cell phones reached the highest values of association and risk among the devices analyzed.

Given this, victims and perpetrators of bullying and cyberbullying show harmful use of the Internet, cell phones, and video games, affecting girls more than boys, and perpetrators are particularly prone to high levels of technological abuse, with cyberbullying being an intensifying factor. It is recommended that strategies and policies be developed to prevent and address bullying and cyberbullying in the educational context by prioritizing student safety. Teachers should provide specialized counseling to the students involved, with an emphasis on girls. Likewise, families should take an active role in digital education, supervise the use of technologies, and work together with schools to ensure a protected educational environment.

## Figures and Tables

**Table 1:** Biometric characteristics; sociodemographic data of participants; average MVPA; bullying and cyberbullying variables; and Internet, cell phone, and video game use segmented by sex.

	All (n = 677)		Boys (n = 318)		Girls (n = 359)		
Variables	Mean	SD	Mean	SD	Mean	SD	*p*
Age (years)	13.81	1.56	13.76	1.67	13.85	1.47	0.452
BMI (kg/m^2^)	20.93	4.12	21.39	4.23	20.53	3.98	0.006
Mother’s level of education (%)							0.047
No studies	37 (5.53)		19 (6.12)		18 (5.01)		
Primary education	95 (14.21)		51 (16.45)		44 (12.25)		
Secondary education	111 (16.59)		46 (14.83)		65 (18.11)		
Professional training	101 (15.09)		47 (15.16)		54 (15.04)		
University studies	218 (32.58)		87 (28.06)		131 (36.49)		
N/C	107 (15.99)		60 (19.35)		47 (13.09)		
Mean MVPA (days/week, ≥1 h)	3.85	1.74	4.08	1.77	3.64	1.7	0.001
Bullying victimization (1–2 a.u.)	1.75	0.76	1.74	0.79	1.75	0.73	0.823
Bullying perpetration (1–2 a.u.)	1.49	0.62	1.58	0.67	1.41	0.56	<0.001
Cyberbullying victimization (1–2 a.u.)	1.28	0.49	1.27	0.47	1.29	0.5	0.665
Cyberbullying perpetration (1–2 a.u.)	1.24	0.50	1.25	0.49	1.23	0.51	0.548
Internet use (1–5 a.u.)	2.33	0.78	2.31	0.78	2.36	0.78	0.409
Cell phone use (1–5 a.u.)	2.59	1.13	2.53	1.09	2.66	1.17	0.137
Video game use (1–5 a.u.)	1.87	0.88	2.16	0.91	1.61	0.76	<0.001

Note: Data are presented as mean for continuous variables and percentage for categorical variables. BMI = body mass index; SD = standard deviation; MVPA = moderate vigorous physical activity. In all cases 1–2: scale 1 = never and 2 = sometimes (had been a victim/offender of bullying and/or cyberbullying). In all cases 1–5: scale 1 = never/totally disagree and 5 = almost always/always/totally disagree, with the highest score being the maximum presence of Internet, smartphone, and video game use addiction in the past year. A. u. = arbitration units.

**Table 2:** Odds ratio (OR) and confidence intervals (95% CI) for levels of victimization/perpetration in bullying and cyberbullying. Internet, cell phone and video game use were included in the logistic regression as a categorical variable (low vs. high). The OR was adjusted for age, body mass index, mother’s educational level and average weekly physical activity.

		All (677)				Boys (318)				Girls (359)			
		N	*p*	OR	95%IC	N	*p*	OR	95%IC	N	*p*	OR	95%IC
**Bullying victimization**													
**Internet**	**Low**	326		1	Referent	151		1	Referent	175		1	Referent
	**High**	341	<0.001	1.606	1.286–2.005	159	0.080	1.313	0.968–1.780	182	<0.001	2.080	1.489–2.906
**Mobile phone**	**Low**	334		1	Referent	169		1	Referent	165			Referent
	**High**	333	<0.001	2.017	1.597–2.548	141	0.098	1.513	1.114–2.054	192	<0.001	2.898	2.000–4.199
**Videogames**	**Low**	344		1	Referent	110		1	Referent	234			Referent
	**High**	323	0.313	1.114	0.901–1.621	200	0.629	1.001	0.911–1.898	123	<0.001	1.767	1.291–2.419
**Bullying perpetration**													
**Internet**	**Low**	326		1	Referent	151		1	Referent	175		1	Referent
	**High**	341	<0.001	2.116	1.576–2.841	159	0.027	1.503	1.047–2.157	182	<0.001	3.826	2.268–6.453
**Mobile phone**	**Low**	334		1	Referent	169		1	Referent	165		1	Referent
	**High**	333	<0.001	2.521	1.850–3.436	141	0.006	1.659	1.158–2.375	192	<0.001	8.068	4.051–16.067
**Videogames**	**Low**	344		1	Referent	110		1	Referent	234		1	Referent
	**High**	323	<0.001	3.002	2.162–4.168	200	0.208	1.181	0.901–2.407	123	<0.001	3.403	2.096–5.526
**Cyberbullying victimization**													
**Internet**	**Low**	326		1	Referent	151		1	Referent	175			Referent
	**High**	341	<0.001	4.531	2.804–7.322	159	0.006	3.279	1.650–6.514	182	<0.001	5.998	3.061–11.752
**Mobile phone**	**Low**	334		1	Referent	169		1	Referent	165			Referent
	**High**	333	<0.001	7.980	4.477–14.222	141	<0.001	4.585	2.223–9.455	192	<0.001	16.473	5.940–45.686
**Videogames**	**Low**	344		1	Referent	110		1	Referent	234			Referent
	**High**	323	<0.001	4.616	2.809–7.583	200	0.049	3.999	1.009–12.732	123	<0.001	5.484	2.967–10.137
**Cyberbullying perpetration**													
**Internet**	**Low**	326		1	Referent	151		1	Referent	175		1	Referent
	**High**	341	<0.001	5.782	3.198–10.455	159	0.019	4.137	1.944–8.803	182	<0.001	8.628	3.282–22.683
**Mobile phone**	**Low**	334		1	Referent	169		1	Referent	165		1	Referent
	**High**	333	<0.001	14.367	6.358–32.465	141	<0.001	8.394	3.377–20.862	192	<0.001	14.333	4.554–38.113
**Videogames**	**Low**	344		1	Referent	110		1	Referent	234		1	Referent
	**High**	323	<0.001	3.839	3.562–13.132	200	<0.001	5.091	1.926–13.459	123	<0.001	9.699	3.676–25.589

## Data Availability

The data presented in this study are available on request from the corresponding author due to privacy of the participants.
