# Correction: Moral-García et al. (2025). Bullying and Cyberbullying Are Associated with Inappropriate Use of the Internet, Cell Phones, and Video Games in Children and Adolescents. *European Journal of Investigation in Health, Psychology and Education*, *15*(5), 82

**DOI:** 10.3390/ejihpe15060108

**Published:** 2025-06-12

**Authors:** José Enrique Moral-García, Alba Rusillo-Magdaleno, Fredy Alonso Patiño-Villada, Emilio J. Martínez-López

**Affiliations:** 1 Department of Didactics of Musical, Plastic and Corporal Expression, Faculty of Humanities and Education Science, University of Jaén, 23071 Jaén, Spain; jemoral@ujaen.es (J.E.M.-G.); emilioml@ujaen.es (E.J.M.-L.); 2 University Institute of Physical Education and Sports, University of Antioquia, Medellín 050010, Colombia; fredy.patino@udea.edu.co

In the original publication (Moral-García et al., 2025), the following paragraphs were mistakenly missed in Results section during a major revision.

A correction has been made to insert the following paragraphs at the end of the Results section.

**Covariance analysis of victimization and perpetration in bullying and cyberbullying with respect to internet use.**

Analysis of covariance employing inappropriate internet use as the dependent variable and bullying measures as the fixed factor showed that, in all cases, victims and perpetrators of bullying, as well as victims and perpetrators of cyberbullying, presented significantly higher scores (10.23%, 13.68%, 11.42% and 19.72%, respectively) of internet abuse compared to all other participants (all F[1,661] > 11.213, *p* < 0.030, ğ > 0.383, Figure 1a–d). When the results were differentiated by sex it was found that, in all cases, girls immersed in bullying situations had significantly higher scores of inappropriate internet use: (A) for bullying victims = 18.72% (2.41 ± 0.78 vs. 2.03 ± 0.73 a.u., F[1,351] = 13.351, *p* < 0.001, ğ = 0.491, 1 − β = 0.954, Figure 1a); (B) for bullying perpetrators = 16.59% (2.46 ± 0.78 vs. 2.11 ± 0.72 a.u., F[1,351] =16.242, *p* < 0.001, ğ = 0.458, 1 − β = 0.980, Figure 1b); (C) for cyberbullying victims = 10.36% (2.45 ± 0.78 vs. 2.22 ± 0.76 a.u., F[1,351] = 14.609, *p* < 0.019, ğ = 0.397, 1 − β = 0.927, Figure 1c) and (D) for cyberbullying perpetrators = 18.43% (2.57 ± 0.76 vs. 2.17 ± 0.75 a.u., F[1,351] = 27.807, *p* < 0.001, ğ = 0.530, 1 − β = 0.999, Figure 1d). For boys, the results showed 21.05% more inappropriate internet use in cyberbullying perpetrators (2.53 ± 0.71 vs. 2.09 ± 0.78 a.u., F[1,304] = 21.894, *p* = 0.033, ğ = 0.5876, 1 − β = 0.967). No significant differences were found in either victims or victims/offenders of bullying (all *p* > 0.05).

**Covariance analysis of victimization and perpetration in bullying and cyberbullying with respect to cell phone use.**

The analysis of covariance employing inappropriate or unhealthy cell phone use as the dependent variable and bullying measures as the fixed factor showed that, in all cases, victims and perpetrators of bullying, as well as victims and perpetrators of cyberbullying, presented higher indicators of cell phone abuse (26.55%, 26.61%, 26.55% and 31.42%, respectively) compared to all other participants (all F[1,661] > 14.363, *p* < 0.009, ğ > 0.358; Figure 2a–d). Results segmented by sex showed that, in all cases, girls involved in bullying contexts had significantly higher scores of inappropriate cell phone use: (A) bullying victims = 29.25% (2.74 ± 1.13 vs. 2.12 ± 1.17 a.u., F[1,351] = 14.171, *p* < 0.001, ğ = 0.539, 1 − β = 956, Figure 2a); (B) bullying perpetrators = 35.85% (2.88 ± 1.11 vs. 2.12 ± 1.16 a.u., F[1,351] = 35.474, *p* < 0.001, ğ = 0.590, 1 − β = 0.999, Figure 2b); (C) cyberbullying victims = 35% (2.97 ± 1.15 vs. 2.2 ± 1.1 a.u., F[1,351] = 56.333, *p* < 0.001, ğ = 0.653, 1 − β = 0.999, Figure 2c) and (D) cyberbullying perpetrators = 33.04% (3.06 ± 1.11 vs. 2.3 ± 1.1 a.u., F[1,351] = 46.874, *p* < 0.001, ğ = 0.611, 1 − β = 0.991, Figure 2d). For their part, boys immersed in bullying situations manifested significantly more inappropriate cell phone use: (A) bullying perpetrators = 16.89% (2.63 ± 1.09 vs. 2.25 ± 1.01 a.u., F[1,304] = 9.497, *p* = 0.011, ğ = 0.357, 1 − β = 0.719, Figure 2b); (B) cyberbullying victims = 14.01% (2.64 ± 1.04 vs. 2.27 ± 1.08 a.u., F[1,304] = 9.753, *p* = 0.016, ğ = 0.337, 1 − β = 0.845, Figure 2c) and (C) cyberbullying perpetrators = 29.28% (2.87 ± 1.09 vs. 2.22 ± 1.01 a.u., F[1,304] = 28.272, *p* < 0.001, ğ = 0.628, 1 − β = 0.979, Figure 2d). However, no significant differences were found in boys who were victims of bullying (*p* > 0.05).

**Covariance analysis of bullying and cyberbullying victimization with respect to video game use.**

Analysis of covariance using inappropriate or unhealthy video game use as the dependent variable and bullying measures as the fixed factor showed that both bullying perpetrators and cyberbullying victims and offenders had higher values of video game abuse (16.87%, 15.20% and 20.71%, respectively) compared to the rest of the participants (all significant: F[1,661] > 13.158, *p* < 0.020, ğ > 0.301; Figure 2b–d). Sex-segmented analysis revealed inappropriate video game use in: (A) bullying perpetrators = 23.19% (1.7 ± 0.82 vs. 1.38 ± 0.53 a.u., F[1,351] = 24.315, *p* < 0.001, ğ = 0.523, 1 − β = 0.945, Figure 3b); (B) cyberbullying victims = 27.54% (1.7 ± 0.82 vs. 1.38 ± 0.53 a.u., F [1,351] = 26.416, *p* < 0.001, ğ = 0.517, 1 − β = 0.979, Figure 3c) and (C) cyberbullying perpetrators = 26.57% (1.81 ± 0.89 vs. 1.43 ± 0.58 a.u., F[1,351] = 27.916, *p* < 0.001, ğ = 0.512, 1 − β = 0.966, Figure 3d). In boys, inappropriate video game use was observed in cyberbullying perpetrators = 16.08% (2.31 ± 0.85 vs. 1.99 ± 0.91 a.u., F[1,304] = 6.424, *p* = 0.012, ğ = 0.362, 1 − β = 0.715, Figure 3d), but not between victims and perpetrators of bullying and victims of cyberbullying (all *p* > 0.05, Figure 3a–c).

**Binary logistic regression on bullying and cyberbullying victimization and perpetration with respect to internet, cell phone and video game use.**

The data showing the risk of exposure to bullying and cyberbullying (victimization and perpetration) with respect to internet, cell phone and video game abuse are presented in Table 2. Overall, bullied schoolchildren were shown to have 1.60 and 2.07 times the risk of inappropriate use of the internet (OR = 1.606; *p* < 0.001) and cell phone (OR = 2.017; *p* < 0.001) than those who were not bullied, respectively. Bullied girls had a higher risk of abusing the internet (OR = 2.080; *p* < 0.001), cell phone (OR = 2.898; *p* < 0.001) and video games (OR = 1.767; *p* < 0.001). On the other hand, bullying perpetrators expressed 2.11, 2.52 and 3 times more risk of inappropriate use of internet, cell phone and video games, respectively (all *p* < 0.001). Both bullying boys and bullying girls were more likely to have unhealthy internet (OR = 1.503; *p* = 0.027 and OR = 3.826; *p* < 0.001, respectively) and cell phone use (OR = 1.659; *p* = 0.006 and OR = 8.068; *p* < 0.001, respectively). On the other hand, the risk of inappropriate use of video games was increased 3.40 times in bullying girls (*p* < 0.001) but not in boys (*p* > 0.05).

The cyberbullying data indicated that cyberbullying victims had a 4.53, 7.98 and 4.61 times higher risk of misusing the internet compared to those who did not suffer cyberbullying. According to gender, in boy and girl victims of cyberbullying, there was a higher probability of abusive use of the internet (OR = 3.279; *p* = 0.006 and OR = 5.998; *p* < 0. 001, respectively), cell phone (OR = 4.585; *p* = < 0.001 and OR = 16.473; *p* < 0.001, respectively) and video games (OR = 3.999; *p* = 0.049 and OR = 5.484; *p* < 0.001, respectively) compared to those who were not bullied. Furthermore, it was detected that schoolchildren perpetrators of cyberbullying had a significant risk of making unhealthy use of internet (OR = 5.782; *p* < 0.001), cell phone (OR = 14.367; *p* < 0.001) and video games (OR = 3.839; *p* < 0.001). This high probability of risk was confirmed in both boy and girl perpetrators with respect to non-perpetrators (all *p* < 0.001).

The authors state that the scientific conclusions are unaffected. This correction was approved by the Academic Editor. The original publication has also been updated.

## Figures and Tables

**Figure 1:**
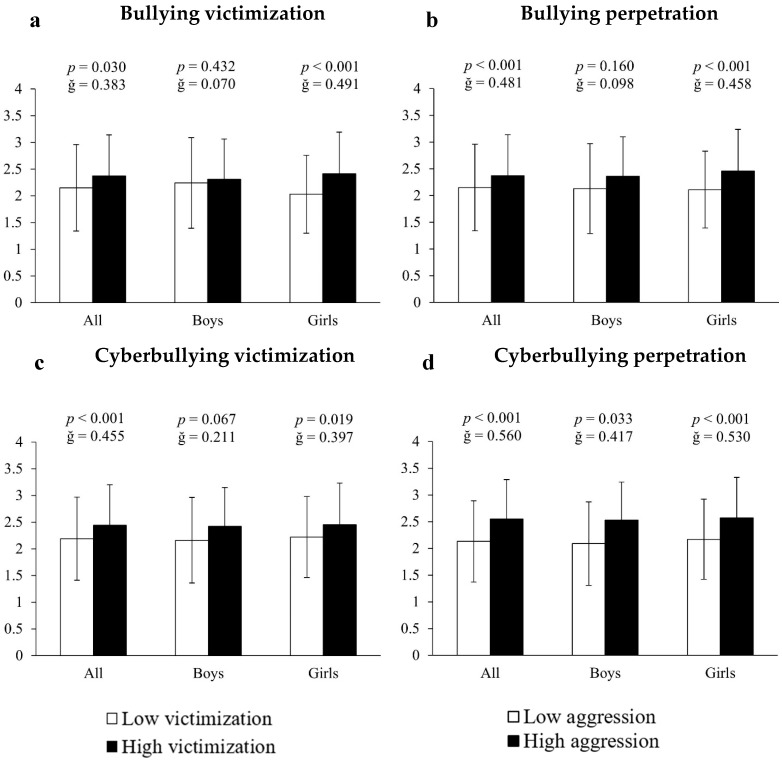
Association of victimization and perpetration in bullying and cyberbullying with respect to internet use.

**Figure 2:**
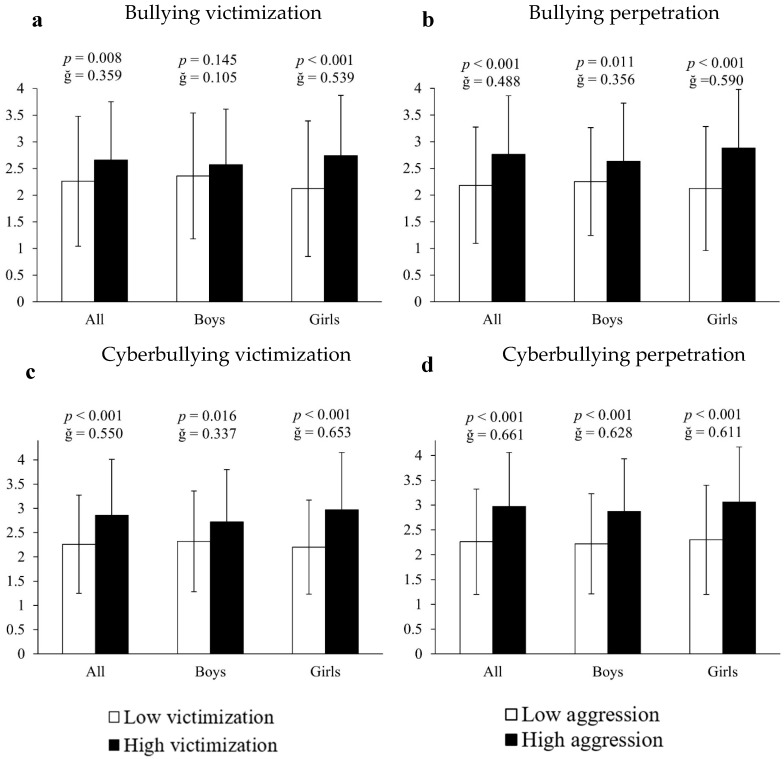
Association of victimization and perpetration in bullying and cyberbullying with respect to cell phone use.

**Figure 3:**
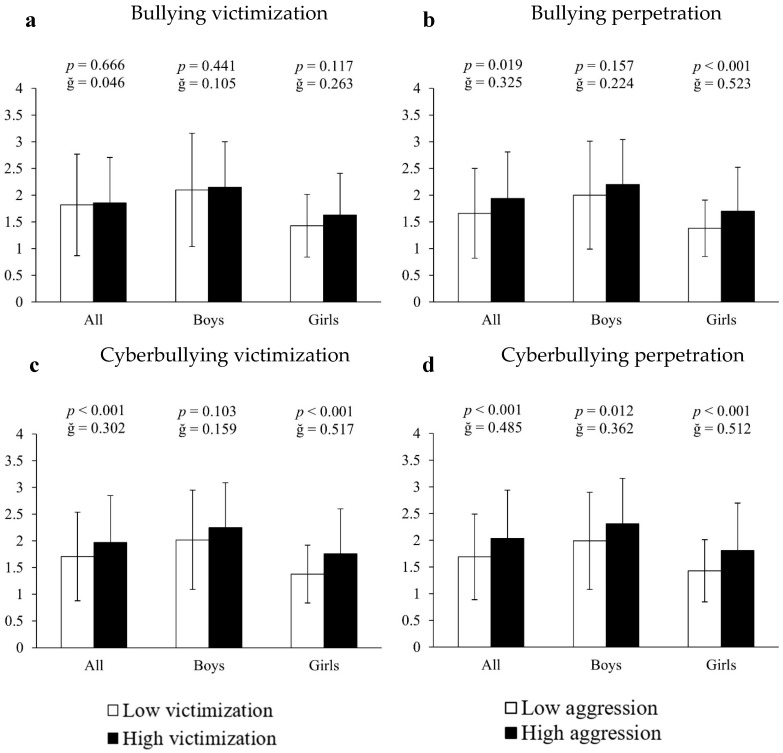
Association of victimization and perpetration in bullying and cyberbullying with respect to the use of video games.

**Table 2:** Odds ratio (OR) and confidence intervals (95% CI) for levels of victimization/perpetration in bullying and cyberbullying. Internet, cell phone and video game use were included in the logistic regression as a categorical variable (low vs. high). The OR was adjusted for age, body mass index, mother’s educational level and average weekly physical activity.

		All (677)				Boys (318)				Girls (359)			
		N	*p*	OR	95%IC	N	*p*	OR	95%IC	N	*p*	OR	95%IC
**Bullying victimization**													
**Internet**	**Low**	326		1	Referent	151		1	Referent	175		1	Referent
	**High**	341	<0.001	1.606	1.286–2.005	159	0.080	1.313	0.968–1.780	182	<0.001	2.080	1.489–2.906
**Mobile phone**	**Low**	334		1	Referent	169		1	Referent	165			Referent
	**High**	333	<0.001	2.017	1.597–2.548	141	0.098	1.513	1.114–2.054	192	<0.001	2.898	2.000–4.199
**Videogames**	**Low**	344		1	Referent	110		1	Referent	234			Referent
	**High**	323	0.313	1.114	0.901–1.621	200	0.629	1.001	0.911–1.898	123	<0.001	1.767	1.291–2.419
**Bullying perpetration**													
**Internet**	**Low**	326		1	Referent	151		1	Referent	175		1	Referent
	**High**	341	<0.001	2.116	1.576–2.841	159	0.027	1.503	1.047–2.157	182	<0.001	3.826	2.268–6.453
**Mobile phone**	**Low**	334		1	Referent	169		1	Referent	165		1	Referent
	**High**	333	<0.001	2.521	1.850–3.436	141	0.006	1.659	1.158–2.375	192	<0.001	8.068	4.051–16.067
**Videogames**	**Low**	344		1	Referent	110		1	Referent	234		1	Referent
	**High**	323	<0.001	3.002	2.162–4.168	200	0.208	1.181	0.901–2.407	123	<0.001	3.403	2.096–5.526
**Cyberbullying victimization**													
**Internet**	**Low**	326		1	Referent	151		1	Referent	175			Referent
	**High**	341	<0.001	4.531	2.804–7.322	159	0.006	3.279	1.650–6.514	182	<0.001	5.998	3.061–11.752
**Mobile phone**	**Low**	334		1	Referent	169		1	Referent	165			Referent
	**High**	333	<0.001	7.980	4.477–14.222	141	<0.001	4.585	2.223–9.455	192	<0.001	16.473	5.940–45.686
**Videogames**	**Low**	344		1	Referent	110		1	Referent	234			Referent
	**High**	323	<0.001	4.616	2.809–7.583	200	0.049	3.999	1.009–12.732	123	<0.001	5.484	2.967–10.137
**Cyberbullying perpetration**													
**Internet**	**Low**	326		1	Referent	151		1	Referent	175		1	Referent
	**High**	341	<0.001	5.782	3.198–10.455	159	0.019	4.137	1.944–8.803	182	<0.001	8.628	3.282–22.683
**Mobile phone**	**Low**	334		1	Referent	169		1	Referent	165		1	Referent
	**High**	333	<0.001	14.367	6.358–32.465	141	<0.001	8.394	3.377–20.862	192	<0.001	14.333	4.554–38.113
**Videogames**	**Low**	344		1	Referent	110		1	Referent	234		1	Referent
	**High**	323	<0.001	3.839	3.562–13.132	200	<0.001	5.091	1.926–13.459	123	<0.001	9.699	3.676–25.589
